# Associations between oral health status and risk of fractures in elder adults

**DOI:** 10.1038/s41598-023-28650-9

**Published:** 2023-01-24

**Authors:** Seok Woo Hong, JunYeop Lee, Jeong-Hyun Kang

**Affiliations:** 1 Department of Orthopedic Surgery, Kangbuk Samsung Hospital, Sungkyunkwan University School of Medicine, 29, Saemunan-Ro, Jongno-Gu, Seoul, 03181 Republic of Korea; 2 Clinic of Oral Medicine and Orofacial Pain, Institute of Oral Health Science, Ajou University School of Medicine, 164, Worldcup-Ro, Yeongtong-Gu, Suwon, Gyeonggi-Do 16499 Republic of Korea

**Keywords:** Endocrinology, Risk factors

## Abstract

Oral health condition, bone mineral density, skeletal muscle mass, fall, fracture, and frailty seem to be strongly interconnected. This study aimed to investigate associations between probability of osteoporotic fractures and oral health in the elderly. In total, 2322 Korean subjects from the 2008—2009 Korea National Health and Nutritional Examination Survey aged over 65 years were included. The 10-year probabilities of major and hip fractures were calculated using the Fracture Risk Assessment (FRAX) tool. Data on anthropometry, skeletal bone mineral density, sociodemographic characteristics, physical activity, individual history of fractures and falls, parental history of osteoporosis, number of teeth, metabolic syndrome, the Decayed, Missing, and Filled permanent Teeth index, and Community Periodontal Index (CPI), were collected. Participants were classified into three groups based on FRAX score for major osteoporotic fractures. A multivariate linear regression analysis was conducted to analyze associations between FRAX scores and oral health-related factors, adjusting for confounding factors. BMI, presence of metabolic syndrome, number of teeth, dental patterns, and CPI score showed significant differences among three groups in males and females. Results from multivariate linear regression analysis demonstrated significant relationships between total tooth number and probabilities of fracture in male and female elderly. The interdisciplinary approach for handling osteoporosis and sarcopenia including dentists, physicians is necessary to facilitate a better quality of life in the elderly.

## Introduction

Aging is accompanied by neuromuscular, immunological, and endocrinological changes in the body that may lead to frailty, featured by weight loss, weakness, slowness, low physical activity, and reduced energy in the elderly^1^. Falls and fractures have been regarded as serious issues in geriatric medicine, as these may lead to frailty, which in turn may result in increased probabilities of falls and fractures^2^. Osteoporosis and sarcopenia are among the most common diseases in the elderly populations and are associated with the increased prevalence of falls and fractures^3,4^. Fall-related fractures can not only influence the quality of life but also lead to higher mortality rates and increased medical and healthcare costs. Therefore, predicting the risk factors for falls and fractures and developing strategies to minimize them in the elder populations are the main concerns of clinicians and policy makers for aged societies.

Oral health condition, bone mineral density (BMD), skeletal muscle mass, and frailty seem to be strongly interconnected^5–22^. The decreased number of teeth has been shown to have significant correlations with the amount of skeletal muscle mass, handgrip strength, and gait speed in the elderly^8,10,17,19^. Furthermore, the link between oral health status and skeletal BMD has also been discussed^7,9,12–16,22^. Finally, poor oral health can result in increased physical frailty, disability, and mortality rate^5,6,11,18,21^. Diverse dental problems in the elderly can result in chewing difficulties which contribute to the changes in food selection that may ultimately result in malnutrition and low bone and muscle mass^20,23^. The influences of poor oral conditions and occlusal support on posture stabilization and balance function, which might cause fall-related events, have been suggested^24^. Though the fragmentary knowledge on the association between oral health and fall- and fracture-related factors, such as BMD, muscle mass, body balance, balancing function, and frailty, has been proposed, thorough and integrated discussions about the relationships among those factors have not been fully elucidated.

Several reports have suggested links between tooth loss and an increased incidence of falls and fractures. One case–control study revealed that the number of missing teeth was related to the probability of hip fractures but this study included a small sample size, which inevitably compromised the significance of the results^25^. Another prospective study showed that individuals with more than 15 missing teeth had a higher risk of hip fractures^26^ and the other study demonstrated the relationships among tooth number, occlusal balance, and the incidence of diverse types of fractures in the elderly^27^. Moreover, one study showed that having 19 or fewer teeth without dentures was associated with a higher risk for incident falls in older adults^28^. However, none of these studies included information on other confounding factors such as skeletal BMD and muscle mass.

Currently, the fracture risk assessment (FRAX), which is a country- and ethnicity-specific instrument developed by the World Health Organization (WHO), is the most widely used fracture prediction tool for adults. In the FRAX, the 10-year probability of both major and hip fractures can be calculated using 10 clinical risk factors^29,30^. The aim of the present study was to investigate the associations between the 10-year probabilities of fracture and oral health-related factors in the Korean population using data from the 2008–2009 Korea National Health and Nutritional Examination Survey (KNHANES) and FRAX algorithm.

## Materials and methods

### Study population

**Figure 1:**
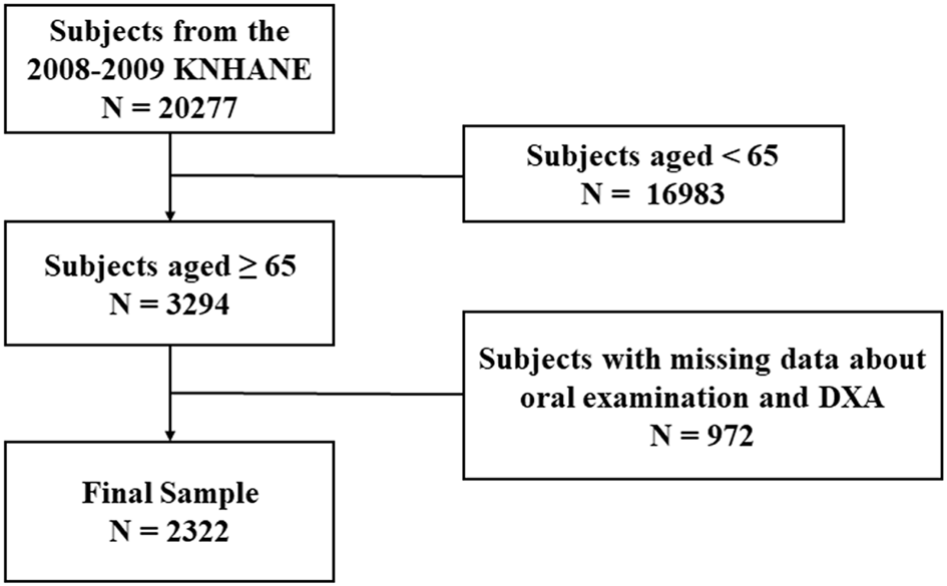
Flow diagram of the study enrollment. KNHANES, Korea National Health and Nutritional Examination Survey; DXA, dual-energy X-ray absorptiometry.

The present study adopted data from the 2008—2009 KNHANES. KNHANES is a nationwide population-based survey conducted by the Korean Center for Disease Control and Prevention. This research included 2322 Korean subjects (959 males, 1363 females) over 65-year old. To include a representative sample of the population, a clustered, multistage, and stratified probability approach was applied. This survey consisted of a nutritional survey, a general health interview, a health assessment, and an oral examination. All participants provided written informed consent following approval from the Institutional Review Board of the Korean Center of Disease Control and Prevention. The ethical approval of this research protocol was exempted by the Institutional Review Board of the tertiary University Hospital (AJIRB-MED-EXP-21–307). Data from eligible participants aged over 65 years were included, and participants with missing data in the health assessments, questionnaires, BMD measurement, and oral examination were excluded (Fig. 1).

### Anthropometric measurements

Qualified staff measured the weight (kg) and height (cm) of all participants. Body mass index (BMI) was calculated by dividing the weight by the square of the height^31^.

### BMD assessments

Whole body dual-energy X-ray absorptiometry (DXA) was applied using a QDR Discovery fan beam densitometer (Hologic, Bedford, MA, USA). The areal BMDs (aBMDs) of the total hip, femoral neck, and lumbar spine and body lean mass were assessed using DXA^32^. Data from DXA were analyzed on the basis of the standard techniques of the Korean Society of Osteoporosis and Hologic Discovery software (version 13.1).

### Sociodemographic parameters, parental history of osteoporosis, and individual history of fractures and falls

The data on sociodemographic data and health behavior such as cigarette smoking, alcohol drinking, levels of physical activity were determined by self-administered survey from KNHANES. The monthly household income and the number of household members were collected for each participant, and the participants were then divided into the following four different categories: < 25% (the lowest quartile group), 25—49%, 50–74%, and 75—100% (the highest quartile group). Education level was also classified into four groups on the basis of the Korean education system: below primary school (≤ 6 years of institutionalized education), middle school (7—9 years of institutionalized education), high school (10—13 years of institutionalized education), and college or higher education (≥ 14 years of institutionalized education).

Cigarette smoking behavior was divided into the following three groups: nonsmokers, smokers who currently smoke and have smoked less than five packs in their entire lives, and smokers who currently smoke and have smoked equal or more than five packs in their entire lives. Alcohol drinking was classified into the following two groups: none or light drinkers (0—3 days/month) and moderate to heavy drinkers (≥ 4 days/month). Physical activity level was measured using the Korean version of the International Physical Activity Questionnaire (IPAQ) short form^33^. The respondents were grouped as performing high/moderate intensity physical activity more than 30 min or over 5 times/week, respectively.

Data on parental history of osteoporosis and individual history of fractures of the spine, hip, or distal radius were collected. Data on individual experiences of falls in the past one year were also included.

### Metabolic syndrome

Diagnosis of metabolic syndrome was performed on the basis of the criteria suggested by previous reports^34,35^. Metabolic syndrome was diagnosed if participants had at least three fulfilling criteria out of the following five criteria: 1) a clinical diagnosis of diabetes treated with oral hypoglycemic medication or insulin or a fasting serum glucose level of 110 mL/dL or higher; 2) arterial blood pressure of 130/85 mm Hg or higher or current use of antihypertensive medication; 3) plasma triglyceride level of 150 mg/dL or higher; 4) high-density lipoprotein cholesterol level of ≤ 50 mg/dL for females or ≤ 40 mg/dL for males; or 5) a waist size greater than 80 cm for females or 90 cm for males.

### Evaluation of the status of oral health

Oral examinations were conducted by trained dentists. Periodontal health status was determined using the Community Periodontal Index (CPI) based on the criteria given by the WHO^36^. CPI scores were categorized as follows: 0, healthy gingiva; 1, gingival bleeding; 2, presence of calculus; 3, pocket depth of 3.5—5.5 mm; and 4, pocket depth of 5.5 mm or more. The ten index teeth were #11, 16, 17, 26, 27, 31, 36, 37, 46, and 47. If no index tooth was present in a sextant qualifying for examination, the adjacent remaining tooth in that sextant was selected. The Decayed, Missing, and Filled permanent Teeth (DMFT) index and the number of teeth present were also measured^37^. Dental patterns including samples with more than 20 teeth, those with teeth number more than 0 but less than 20, and edentulous were also determined.

### Fracture probability calculations

The 10-year probabilities of major and hip fractures were calculated using the FRAX tool (version 3.7, South Korean model), which calculated the probability based on age, sex, aBMD of the femoral neck, individual history of fractures, parental history of hip fractures, current tobacco use, rheumatoid arthritis, alcohol consumption (more than three units per day), secondary osteoporosis, and long-term use of glucocorticoids^29^. Data on individual histories of fractures, current tobacco use, rheumatoid arthritis, alcohol consumption, and secondary osteoporosis were collected from the health interview surveys. As the 2008—2009 KNHANES did not include data regarding the long-term use of glucocorticoids or parental history of hip fractures, negative answers were entered into the FRAX tool as in previous reports^38–40^.

### Statistical analysis

Statistical analyses were performed on the basis of a complex design including stratification, clustering, and weighting. Sample weights were constructed for the sample participants to represent the Korean population by accounting for the survey nonresponses, complex survey design, and stratification (according to age, sex, and geographic area).

All analyses were conducted separately for males and females owing to the different amounts of skeletal muscle mass and background bone metabolism rates between the sexes. The participants were classified into three groups based on the 10-year probable risk of major fracture^41^. The participants whose FRAX scores were less than 10% were classified as Low-Risk group (10% > FRAX score), whose FRAX scores between 10 to 20% were as Moderate-Risk group (20 > FRAX score ≥ 10%), and whose FRAX scores above 20% were as High-Risk group (FRAX score ≥ 20%). Independent t-tests and Rao-Scott chi-square tests were applied to compare the differences in the sociodemographic, anthropometric, and behavioral factors, the number of teeth, DMFT, CPI, 10-year probabilities of major and hip fractures, lean body mass, and skeletal BMD for continuous and categorical variables, respectively. Multivariate linear regression analysis was applied to analyze the associations between the 10- year probabilities of major or hip fractures and oral health-related factors, adjusted for the potential confounders including lean body mass, presence of metabolic syndrome, parental history of osteoporosis, and recent experience of falls. Each anthropometric and sociodemographic variable with a significant association with FRAX scores in the univariate analysis was integrated into the multivariate linear regression analysis to identify interdependent contributions. The variables which included in calculation of FRAX score such as age, aBMD of the femoral neck, individual history of fractures, current smoking, and alcohol consumption were excluded in multivariate analysis. Finally, the independent variables were total teeth number, DMFT, CPI, lean body mass, incidence of metabolic syndrome, history of fall, and parental osteoporotic history and the outcome variable were major fracture probability and hip fracture probability.

### Ethical approval and informed to consent

Written informed consents were obtained from all participants following approval from the Institutional Review Board of the Korean Center of Disease Control and Prevention. The ethical approval of this research protocol was exempt by the Institutional Review Board of the University Hospital (AJIRB-MED-EXP-21–307).

## Results

No significant differences in the levels of household income and education, smoking status, alcohol consumption, the intensity of physical activities, parental history of osteoporosis, individual history of recent experience of falls, and history of fracture of the lumbar spine, hip, or distal radius were detected among the three groups in both male and female elderly. Significant differences in BMI, existence of metabolic syndrome, number of present teeth, dental patterns, and CPI score were observed for both males and females, whereas the DMFT showed significant differences among groups only in the female elderly. There were significant differences in the aBMDs of the femoral neck among the three groups in males. Otherwise, the aBMD of the lumbar spine and lean body mass showed significant differences among the three groups in the female elderly (Tables 1, 2).

**Table 1:** Differences of sociodemographic characteristics and levels of physical activity accordance with the levels of probable risk of major fracture.

Risk of major fracture								
Variable	Total N	Low risk (10% > FRAX)		Moderate risk (20 > FRAX ≥ 10%)		High risk (FRAX ≥ 20%)		*P* value
		N	Mean ± SE or % (95% CI)	N	Mean ± SE or % (95% CI)	N	Mean ± SE or % (95% CI)	
**Male**								
Age† (years) (Mean ± SE)	957	799	71.8 ± 0.2	120	73.0 ± 0.5	38	71.2 ± 0.9	0.061
BMI† (kg/m^2^) (Mean ± SE)	954	797	22.6 ± 0.1	119	24.4 ± 0.3	38	24.4 ± 0.7	< 0.001**
Household income	937							0.058
< 25%		391	49.7 (45.5–54.0)	59	53.4 (43.5–63.0)	13	33.3 (18.0–53.2)	
25–49%		218	27.9 (24.3–31.7)	31	26.3 (18.5–36.0)	8	27.1 (12.3–49.5)	
50–74%		105	13.2 (10.6–16.4)	17	15.8 (9.3–25.6)	9	26.5 (13.0–46.4)	
≥ 75%		72	9.1 (7.0–11.9)	8	4.5 (2.1–9.4)	6	13.2 (6.2–25.9)	
Education	946							0.117
≤ primary school		447	54.7 (50.0–59.3)	60	46.8 (36.3–57.6)	15	38.7 (21.2–59.7)	
Middle school		137	16.5 (13.7–19.6)	21	20.3 (13.0–30.4)	6	13.2 (6.7–24.2)	
High school		127	18.1 (14.9–21.8)	19	16.3 (9.4–26.9)	12	32.2 (16.8–52.8)	
≥ College or higher		79	10.8 (8.5–13.7)	18	16.5 (9.5–27.1)	5	15.9 (5.3–38.7)	
Smoking status	950							0.311
Never of former		8	1.1 (0.5–2.1)	2	1.2 (0.2–6.1)	0	0	
≤ 5 packs		657	80.9 (77.1–84.1)	89	73.4 (62.8–81.8)	31	81.8 (65.9–91.2)	
≥ 5 packs		129	18.0 (14.7–21.5)	27	25.4 (17.1–35.9)	7	18.2 (8.8–34.1)	
Alcohol consumption	951							0.072
None or light		98	12.2 (9.5–15.3)	12	9.6 (4.9–18.0)	7	27.2 (13.3–47.6)	
Moderate or heavy		697	87.8 (84.5–90.2)	106	90.4 (82.0–95.1)	31	72.8 (52.4–86.7)	
High intensity physical activity	950							0.298
Yes		105	12.7 (10.1–15.9)	11	9.0 (4.8–16.4)	7	21.3 (7.9–45.9)	
No		690	87.3 (84.1–89.9)	106	91.0 (83.6–95.2)	31	78.7 (54.1–92.1)	
Moderate intensity physical activity	949							0.063
Yes		124	15.6 (12.6–19.2)		7.8 (4.3–13.8)		20.7 (8.5–42.2)	
No		670	84.4 (80.8–87.4)		92.2 (86.2–95.7)		79.3 (57.8–91.5)	
Metabolic Syndrome	957							0.030*
Yes		231	28.1 (24.7–31.8)	51	42.0 (32.0–52.8)	15	33.5 (18.3–53.1)	
No		568	71.9 (68.2–75.3)	69	58.0 (47.2–68.0)	23	66.5 (46.9–81.7)	
**Female**								
Age† (years) (Mean ± SE)	1363	711	72.4 ± 0.2	462	71.6 ± 0.2	190	72.2 ± 0.4	0.038*
BMI† (kg/m^2^) (Mean ± SE)	1359	708	23.4 ± 0.2	461	24.6 ± 0.2	190	24.8 ± 0.2	< 0.001**
Household income	1321							0.798
< 25%		395	58.1 (53.3–62.8)	252	56.9(51.7–61.9)	115	60.9(52.2–68.9)	
25–49%		146	20.7 (17.3–24.5)	100	22.5(18.7–26.9)	35	21.1(15.0–28.7)	
50–74%		82	12.3 (9.6–15.6)	57	13.3(10.2–17.2)	17	7.6(4.6–12.4)	
≥ 75%		64	8.9 (6.5–12.1)	39	7.3(5.0–10.5)	19	10.4(6.6–16.2)	
Education	1347							0.103
≤ primary school		641	91.4(88.5–93.5)	409	90.0(86.4–92.7)	160	86.7(80.0–91.4)	
Middle school		30	4.3(2.8–6.5)	26	5.8(3.8–8.9)	13	6.7(3.7–11.9)	
High school		21	2.7(1.7–4.5)	19	4.0(2.5–6.4)	10	4.6(2.3–8.9)	
≥ College or higher		13	1.7(0.9–3.1)	1	0.2(0–1.3)	4	2.1(0.7–5.6)	
Smoking status	1346							0.365
Never of former		7	1.5 (0.5–3.5)	4	0.9(0.3–2.3)	11	1.5 (0.2–7.5)	
≤ 5 packs		78	11.7 (8.8–14.8)	46	10.1(7.3–13.4)	24	13.3(8.4–19.0)	
≥ 5 packs		620	86.8 (83.1–89.5)	407	89.0(0–1.6)	159	85.2(77.5–89.7)	
Alcohol consumption	1348							0.076
None or light		344	49.9(45.7–53.9)	222	48.0(42.2–53.7)	82	44.3(36.5–51.3)	
Moderate or heavy		362	50.1(45.9–54.1)	235	52.0(46.1–57.6)	103	55.7(47.8–62.6)	
High intensity physical activity	1347							0.116
Yes		73	11.3 (8.3–15.2)	37	7.3 (5.0–10.7)	17	7.7 (4.5–13.1)	
No		633	88.7 (84.8–91.7)	420	92.7 (89.3–95.0)	167	92.3 (86.9–95.5)	
Moderate intensity physical activity	1345							0.553
Yes		107	15.5 (12.4–19.3)	60	13.2 (9.9–17.5)	23	13.0 (8.5–19.3)	
No		597	84.5 (80.7–87.6)	397	86.8 (82.5–90.1)	161	87.0 (80.7–91.5)	
Metabolic Syndrome	1359							0.023*
Yes		357	49.8 (45.2–54.3)	273	58.5 (53.3–63.5)	118	56.2 (48.1–63.9)	
No		352	50.2 (45.7–54.8)	187	41.5 (36.5–46.7)	72	43.8 (36.1–51.9)	

BMI, body mass index; CI, confidential interval; SE, standard error.

%Weighted percentage by column.

Data obtained from Rao-Scott Chi-square test.

^†^Data obtained from independent T-test and descriptive values are shown as mean ± SE.

*P < 0.05, **P < 0.001 by Chi-Square test or independent T-test.

**Table 2:** Differences of oral health status, history related with fracture risk, bone mineral density, lean body mass according to the levels of probable risk of major fracture.

Risk of major fracture								
Variable	Total N	Low risk (10% > FRAX)		Moderate risk (20 > FRAX ≥ 10%)		High risk (FRAX ≥ 20%)		*P* value
		N	Mean ± SE or % (95% CI)	N	Mean ± SE or % (95% CI)	N	Mean ± SE or % (95% CI)	
**Male**								
Number of teeth	957	799	16.3 ± 0.4	120	12.2 ± 0.9	38	11.7 ± 1.3	< 0.001**
Dental pattern^†^								< 0.001**
> 20		338	42.5 (38.5–46.6)	34	14.0 (8.8–21.6)	7	16.8 (6.5–36.8)	
0 < Teeth No ≤ 20		375	46.9 (42.8–51.0)	66	60.4 (49.9–70.0)	21	62.1 (44.7–76.9)	
Edentulous		86	10.6 (8.6–13.1)	20	25.6 (17.5–35.9)	10	21.1 (10.9–36.9)	
DMFT	957	799	9.98 ± 0.32	120	11.6 ± 0.8	38	8.34 ± 1.30	0.074
CPI^†^	957							0.004*
0		192	23.3 (20.5–26.5)	41	33.1 (24.6–43.0)	17	35.3 (23.0–49.9)	
1		56	7.0 (5.4–9.1)	8	6.1 (2.6–13.4)	2	12.5 (3.3–37.4)	
2		188	23.3 (19.9–27.0)	30	23.1 (15.3–33.5)	9	27.2 (12.8–48.8)	
3		301	38.2 (34.4–42.2)	36	33.3 (24.0–44.0)	9	21.1 (9.6–40.2)	
4		62	8.1 (6.1–10.8)	5	4.4 (1.6–11.6)	1	4.0 (0.6–23.4)	
10 year probability (%)	946	788		120		38		
Major fracture			4.54 ± 0.94		14.0 ± 0.3		23.1 ± 0.4	< 0.001**
Hip fracture			1.91 ± 0.56		7.79 ± 0.32		14.4 ± 0.5	< 0.001**
Parental osteoporosis history	953							0.780
Yes		69	9.0 (6.5–11.3)	10	6.7 (3.3–13.2)	1	4.9 (0.7–28.1)	
No		726	91.0 (88.0–92.9)	110	93.3 (86.8–96.7)	37	95.1 (71.9–99.3)	
Recent history of falls	953							0.893
Yes		92	11.5 (8.7–14.3)	12	12.3 (6.1–23.3)	4	10.9 (3.8–27.5)	
No		703	88.5 (84.9–90.8)	108	87.7 (76.7–93.9)	34	89.1 (72.5–96.2)	
Individual fracture history	955							0.971
None		794	99.4 (98.6–99.8)	118	100	38	100	
Yes		5	0.6 (0.2–1.4)	0	0	0	0	
Total hip aBMD (g/cm^2^)	949	791	1.000 ± 0.001	120	1.000 ± 0.001	38	0.992 ± 0.005	0.142
Femur neck aBMD (g/cm^2^)	949	791	1.000 ± 0.001	120	0.968 ± 0.018	38	0.960 ± 0.009	< 0.001**
L1-4 aBMD (g/cm^2^)	916	767	0.998 ± 0.006	113	1.007 ± 0.007	36	1.000 ± 0.001	0.692
Lean body mass (kg)	957	799	43.2 ± 0.2	120	43.7 ± 0.6	38	43.9 ± 1.9	0.737
**Female**								
Number of teeth	1363	711	16.9 ± 0.4	462	15.4 ± 0.5	190	12.8 ± 0.7	< 0.001**
Dental pattern^†^								< 0.001**
> 20		347	48.8 (44.8–52.9)	62	12.0 (9.2–15.5)	29	15.3 (10.0–22.6)	
0 < Teeth No ≤ 20		299	42.6 (38.5–46.8)	221	47.2 (41.8–52.7)	110	58.7 (51.0–66.0)	
Edentulous		65	8.5 (6.6–11.1)	177	40.8 (35.3–46.5)	51	26.0 (19.9–33.2)	
DMFT	1363	711	11.9 ± 0.4	462	10.9 ± 0.4	190	9.68 ± 0.55	< 0.001**
CPI^†^	1359							0.002*
0		163	21.9 (18.5–25.8)	132	27.4 (23.1–32.1)	60	32.9 (25.7–41.0)	
1		44	6.3 (4.6–8.5)	31	7.3 (5.0–10.5)	23	12.2 (7.7–18.9)	
2		196	28.0 (24.7–31.6)	128	27.4 (23.2–32.0)	44	24.0 (17.8–31.5)	
3		250	36.4 (32.2–40.8)	148	33.4 (28.6–38.6)	55	25.9 (20.4–32.3)	
4		56	7.4 (5.5–9.8)	21	4.6 (2.7–7.9)	8	5.0 (2.5–10.1)	
10 year probability	1350	698		462		190		
Major fracture			6.32 ± 0.10		12.7 ± 0.2		25.5 ± 0.5	< 0.001**
Hip fracture			2.20 ± 0.60		5.52 ± 0.14		16.2 ± 0.5	< 0.001**
Parental osteoporosis history	1357							0.799
Yes		98	14.2 (11.2–17.3)	60	14.6 (10.9–19.4)	25	13.3(8.6–20.0)	
No		609	85.6 (82.2–88.4)	400	85.4 (80.6–89.1)	165	86.7(80.0–91.4)	
Recent history of falls	1358							0.925
Yes		128	17.6 (14.4–21.1)	96	21.6 (17.2–26.8)	40	19.3(14.1–26.0)	
No		580	82.4 (78.7–85.4)	364	78.4(73.2–82.8)	150	80.7(74.0–85.9)	
Individual fracture history	1338							0.909
None		668	94.4 (91.5–95.6)	434	95.0 (91.1–96.2)	180	95.2 (88.9–97.5)	
Yes		32	5.6 (3.2–6.7)	17	5.0 (2.4–6.5)	7	4.5 (1.9–9.4)	
Menopause age	1109	567	48.1 ± 0.3	381	48.4 ± 0.3	161	48.5 ± 0.4	0.531
Total hip aBMD (g/cm^2^)	1354	704	0.993 ± 0.007	461	0.981 ± 0.008	189	0.982 ± 0.005	0.361
Femoral neck aBMD (g/cm^2^)	1354	704	0.767 ± 0.032	461	0.751 ± 0.022	189	0.699 ± 0.021	0.100
L1-4 aBMD (g/cm^2^)	1318	683	0.985 ± 0.011	450	0.995 ± 0.004	185	0.959 ± 0.010	0.003*
Lean body mass (kg)	1363	711	32. 3 ± 0.3	462	32.2 ± 0.2	1363	31.6 ± 0.2	0.015*

aBMD, areal bone mineral density; CI, confidential interval; CPI, community periodontal index; DMFT, decay, missing, filling tooth; SE, standard error.

%Weighted percentage by column.

Data obtained from independent T-test.

Descriptive values are shown as mean ± SE.

^†^Data obtained from Rao-Scott Chi-square test and descriptive values are show as % (95% CI).

*P < 0.05, **P < 0.001 by independent T-test and Rao-Scott Chi-square test.

The results from the multivariate linear regression analysis demonstrated significant relationships between total teeth number and 10-year major and hip fracture probabilities in both male and female elderly. Lean body mass did not show significant associations with FRAX score in both male and female elderly. On the other hand, the individual’s recent experience of falls interacted with the probabilities of both major and hip fractures only in the female elderly but not in the male elderly (Table 3).

**Table 3:** Adjusted association between ten-year probability major fracture and hip fracture and oral health related factors.

Male	Major fracture probability (R^2^ = 0.054, *P* < 0.001)		Hip fracture probability (R^2^ = 0.051 *P* = 0.002)	
	B (95% CI)	*P* value	B (95% CI)	*P* value
Total tooth number	− 0.087 (− 0.135 to − 0.040)	< 0.001**	− 0.065 (− 0.097 to − 0.033)	< 0.001**
DMFT	0.005 (− 0.056 to 0.065)	0.881	− 0.001 (− 0.042 to 0.039)	0.944
Lean body mass	0.002 (− 0.090 to 0.093)	0.974	− 0.015 (− 0.071 to 0.042)	0.610
Metabolic syndrome				
Yes	− 1.359 (− 2.368 to − 0.350)	0.009*	− 0.633 (− 1.307 to 0.040)	0.065
No	Reference		Reference	
Recent history of falls				
Yes	− 1.132 (− 3.264 to 1.000)	0.297	0.144 (− 1.013 to 1.302)	0.806
No	Reference		Reference	
Parental osteoporosis history				
Yes	− 0.324 (− 1.571 to 0.922)	0.609	− 0.215 (− 1.012 to 0.582)	0.596
No	Reference		Reference	
CPI				
0	Reference		Reference	
1	0.001 (− 2.108 to 2.107)	0.987	− 0.228 (− 1.545 to 1.088)	0.733
2	− 0.161 (− 1.424 to 1.102)	0.802	− 0.086 (− 0.975 to 0.803)	0.849
3	− 0.919 (− 1.939 to 0.101)	0.077	− 0.561 (− 1.231 to 0.108)	0.100
4	− 0.628 (− 2.037 to 0.780)	0.380	− 0.564 (− 1.532 to 0.404)	0.252

Female	Major fracture probability (R^2^ = 0.066, *P* = 0.004)		Hip fracture probability (R^2^ = 0.058, *P* = 0.009)	
	B (95% CI)	*P* value	B (95% CI)	*P* value
Total tooth number	− 0.113 (− 0.170 to − 0.056)	< 0.001**	− 0.086 (− 0.129 to − 0.044)	< 0.001**
DMFT	− 0.088 (− 0.152 to − 0.024)	0.008*	− 0.057 (− 0.105 to − 0.010)	0.018*
Lean body mass	0.093 (− 0.024 to 0.211)	0.119	0.041 (− 0.048 to 0.130)	0.362
Menopause age	0.030 (− 0.046 to 0.105)	0.778	0.002 (− 0.057 to 0.061)	0.951
Metabolic syndrome				
Yes	− 1.012 (− 1.999 to − 0.025)	0.045*	− 0.444 (− 1.185 to 0.297)	0.239
No	Reference		Reference	
Recent history of falls				
Yes	5.888 (4.740 to 7.037)	< 0.001**	2.587 (1.764 to 3.410)	< 0.001**
No	Reference		Reference	
Parental osteoporosis history				
Yes	− 0.428 (− 1.603 to 0.748)	0.474	− 0.522 (− 1.351 to 0.308)	0.217
No	Reference		Reference	
CPI				
0	Reference		Reference	
1	2.435 (− 0.070 to 4.941)	0.057	1.788 (− 0.449 to 4.024)	0.117
2	0.120 (− 1.496 to 1.735)	0.884	0.130 (− 1.041 to 1.300)	0.827
3	− 0.371 (− 1.716 to 0.973)	0.587	− 0.380 (− 1.329 to 0.568)	0.430
4	− 0.982 (− 3.192 to 1.228)	0.382	− 0.324 (− 1.961 to 1.313)	0.697

CI, confidential interval; CPI, community periodontal index; DMFT, decay, missing, filling tooth.

Data obtained from the multivariate linear regression.

*P < 0.05, **P < 0.001 by the multivariate linear regression.

## Discussion

The prevention of falls and fractures has been regarded as one of the main issues in aged societies because those may lead to frailty and increased medical and social burdens. Many efforts have been attempted to determine the risk factors for falls and fractures. Previous studies have demonstrated the strong relationships among oral health-related factors, the incidence of falls, amount of skeletal muscle mass, frailty, skeletal BMD, physical performance, and mortality rate in the elderly population^5–22,27^. Even though, the fragmentary associations between those factors have been reported previously, the integrated knowledge of interactions among the above factors has not been thoroughly discussed. Hence, the purpose of the present study was to investigate the association between the probabilities of major and hip fractures and oral health related factors, muscle mass, and skeletal BMD in the elder Korean population aged over 65 years using data from the KNHANES and FRAX algorithm.

The novel finding of the present study was the strong link between the probabilities of fractures and the number of teeth present in the elderly. The aforementioned results from the multivariate linear regression analysis demonstrated that the number of teeth present showed significant associations with the 10-year probabilities of major and hip fractures in both male and female elderly after adjusting for confounding factors. Losing teeth in the elderly may lead to chewing difficulty, contributing to the changes in food selection, malnutrition, and low bone and muscle mass^20,23^. Moreover, several previous studies have proposed the role of remaining teeth, bilateral occlusal support, and proprioception from periodontal ligaments on postural balance and incidence of falls^23,24,27^. Although, the precise occlusal condition could not be derived from KNHANES data, the role the remaining teeth on the risk of fracture, including keeping nutritional balance which has associations with bone and muscle mass and body balancing capability which may lead to the occurrence of falls, could be assumed.

Even though the CPI scores showed significant interactions with risk of osteoporotic fractures in univariate analysis, the elderly in high risk group did not show more severe periodontal health status than elderly in low and moderate risk group. Owing to the calculating method of CPI index, the individuals with higher number of teeth loss could have less CPI scores than those with higher remaining teeth with periodontitis or gingivitis. The results from the present study could carefully suggest that the role of number of remaining teeth in the oral cavity would be more important than the inflammatory status in periodontium on incidence of osteoporotic fracture in elderly.

One interesting point was that significant associations between the individual history of falls and FRAX scores were detected in the female elderly only. Generally, the female elderly has lower skeletal BMD and fragile microstructures of bone compared to male elderly due to hormonal changes. Therefore, the relative impact of fall down including osteoporotic fracture would be much higher in female elderly than those in male elderly with less fragile bony structure.

To the best of our knowledge, the present study is the first study to reveal the relationships between the probabilities of fractures and oral health status after adjusting for factors related to osteoporosis and sarcopenia with sufficient sample size. However, this study has several limitations. First of all, due to the cross-sectional study design, the causal relationships between oral health status and actual fracture risk could not be revealed. Secondly, lack of information about long-term glucocorticoid use and parental hip fracture history in the 2008–2009 KNHANES databases could have led to distorted results being derived from the FRAX tool. Despite these limitations, this study still has value for clinicians and policy makers, that studies with a relatively large size of samples from an authorized institution with a proper study design could provide valid and meaningful results.

Good oral health influences the quality of life in a myriad way, including social interactions, communications, self-esteem, resilience, and adequate nutritional intake. Elder adults with oral health problems may experience social isolation and functional decline. Fractures in the elderly are a complex process determined by a combination of diverse factors. As the tooth number in the elderly is an indicator of physical, mental, and social well-being, it can be considered as a potential parameter of fracture risk in elder population. Hence, the oral health related factors, particularly the number of remaining teeth, can have strong associations with fracture probabilities in elder populations. A comprehensive understanding of the necessities of oral healthcare is warranted for dentists, physicians, and even policy makers to facilitate a better quality of life in the elderly and saving social and medical costs in aged society. From this study, deriving social consensus of necessities of interdisciplinary approaches for managing falls and fracture including oral care could be expected.

## Data Availability

The datasets used and/or analyzed during the current study are available from the homepage of Korean Center for Disease Control and Prevention (http://knhanes.kdca.go.kr) with no restriction apply to the availability of these data.
